# Postoperative Cholestasis After Uncomplicated Laparoscopic Cholecystectomy: A Case Report

**DOI:** 10.1155/cris/9983406

**Published:** 2026-09-03

**Authors:** Sarah Y. Cao, Damien J. Lazar

**Affiliations:** ^1^ Department of Surgery, Icahn School of Medicine at Mount Sinai, New York, New York, USA, mountsinai.org

**Keywords:** benign postoperative cholestasis, cholecystectomy, postoperative jaundice

## Abstract

**Introduction:**

In the absence of complications, transient jaundice has been observed in postoperative patients after abdominal surgery. This jaundice has been previously attributed to intrahepatic cholestasis due to oxidative stress from surgery and can be marked within the first postoperative weeks. However, this change has often been seen in patients with significant comorbidities. This report describes the case of a young, healthy patient who developed severe postoperative jaundice after a routine laparoscopic cholecystectomy with no evidence of biliary injury or obstruction, leading to endoscopic retrograde pancreatography and severe necrotizing pancreatitis.

**Case Presentation:**

A 45‐year‐old male with no medical comorbidities presented to our hospital with signs and symptoms consistent with acute cholecystitis. He promptly underwent an uncomplicated laparoscopic cholecystectomy with routine intraoperative cholangiogram (IOC) that was unrevealing. He represented on postoperative day two to the emergency department with a severe conjugated hyperbilirubinemia with unremarkable cross‐sectional imaging. He subsequently underwent endoscopic retrograde pancreatography without evidence of biliary obstruction or biliary leak, after which his hyperbilirubinemia resolved without any further intervention over the following days. As a result of the endoscopic retrograde pancreatography, he developed a complicated case of necrotizing pancreatitis, with a course including multiple operative interventions.

**Conclusion:**

In patients with severe postoperative jaundice, postoperative intrahepatic cholestasis should be considered on the differential in the absence of evidence of other surgical complication. Surgeons should be aware of potential benign causes of hyperbilirubinemia. After laparoscopic cholecystectomy, many important complications are diagnosed and treated with endoscopic retrograde pancreatography. Given its associated potential morbidity, pursuing biliary imaging should be a consideration.

## 1. Introduction

First described in the 1960s, transient jaundice has been observed in postoperative patients after abdominal surgery in the absence of complications [1]. Bilirubin levels can become markedly elevated within the first postoperative weeks, believed to be due to cholestasis caused by operative oxidative stress on hepatocytes [2, 3]. However, these cases have primarily been described in patients with hepatic or cardiac comorbidities. This report describes the case of a healthy young patient with no medical comorbidities who underwent routine laparoscopic cholecystectomy and developed severe postoperative jaundice, ultimately leading to endoscopic retrograde pancreatography and severe complications thereof.

## 2. Case Presentation

The patient was a 45‐year‐old healthy male who presented to the emergency department with 1 day of postprandial right upper quadrant pain in the setting of 1 month of worsening intermittent pain. Upon workup, he was noted to have right upper quadrant abdominal tenderness, leukocytosis, and abdominal ultrasound demonstrating cholelithiasis with gallbladder wall thickening and pericholecystic fluid, all consistent with a diagnosis of acute cholecystitis. The patient’s hepatic function panel, lipase, and common bile duct diameter were all within normal limits, with the duct noted to be 5 mm.

**Figure 1:**
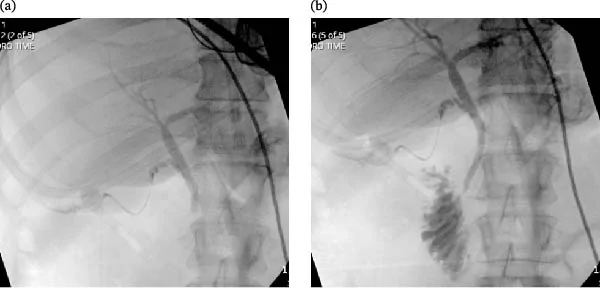
Intraoperative cholangiogram. (a) Demonstrated lack of initial flow of contrast into the duodenum. (b) Contrast flow into duodenum after successful flushing of cholangiogram catheter.

He was started on an appropriate antibiotic regimen and promptly brought to the operating room for an uncomplicated laparoscopic cholecystectomy. Routine intraoperative cholangiogram (IOC) was performed per surgeon preference. After an adequate critical view of safety was achieved, the cystic duct was clipped and a small ductotomy was made in the distal cystic duct proximal to the clip. A cholangiogram catheter was introduced to the abdomen in the right upper quadrant, and the tip was secured in the duct with a clip. Saline was flushed into the duct under visualization, ensuring that the catheter was adequately positioned with no backflow from the ductotomy. While on fluoroscopy there was initially no flow of contrast into the duodenum, after flushing the duct with saline, repeat cholangiogram demonstrated free flow of contrast into the duodenum with normal biliary anatomy. A representative image is shown in Figure 1. The remainder of the cholecystectomy was completed without complication. Postoperatively, he was discharged home after recovery.

On postoperative day 2, the patient was sent to the emergency room for evaluation after noting progressive jaundice and darkening of the urine. On presentation to the emergency room, he was found to have a significantly elevated bilirubin level. Vital signs were within normal limits and physical exam was otherwise consistent with a normal postoperative state. CT abdomen/pelvis was unrevealing. No intrahepatic or extrahepatic ductal dilation was noted.

**Figure 2:**
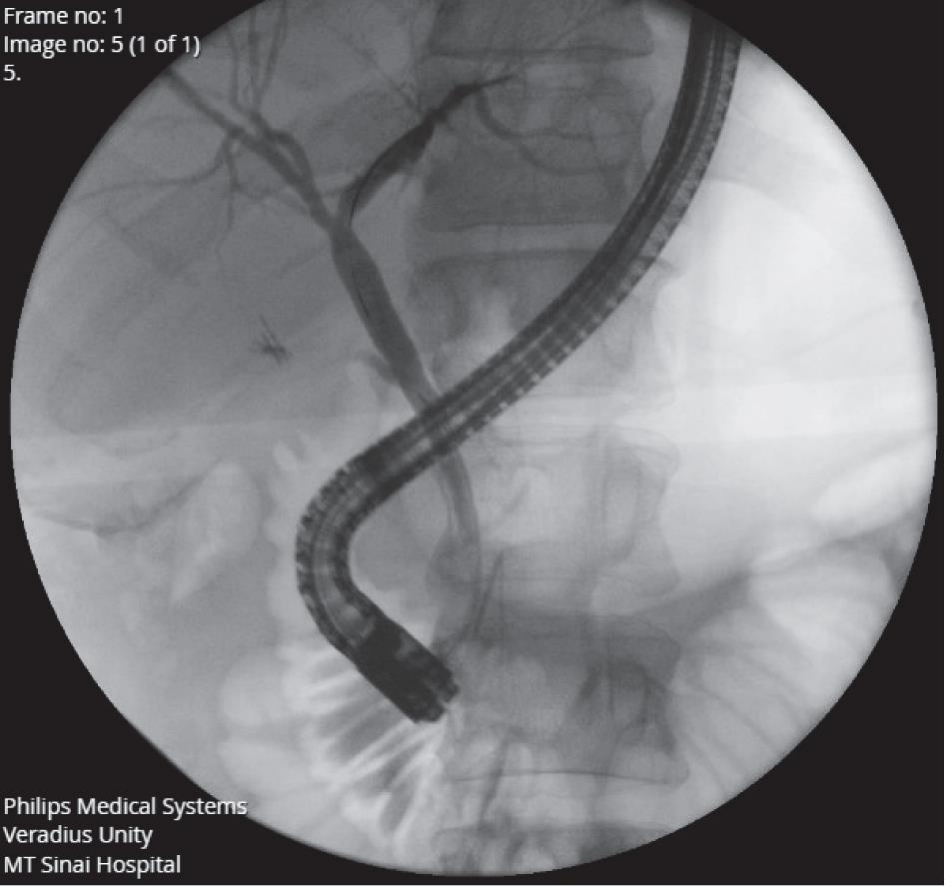
Initial image of ERCP.

**Figure 3:**
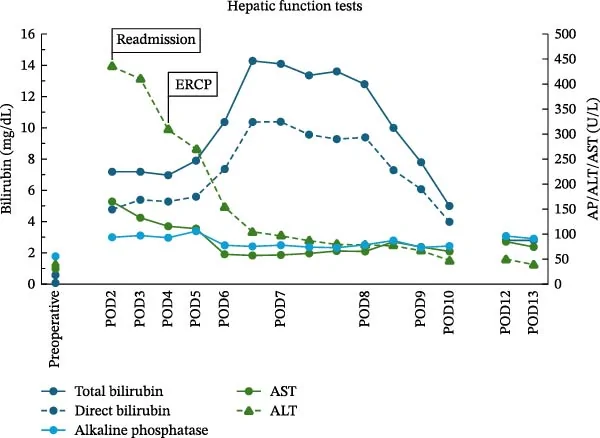
Hepatic function panel throughout the initial hospital course.

**Figure 4:**
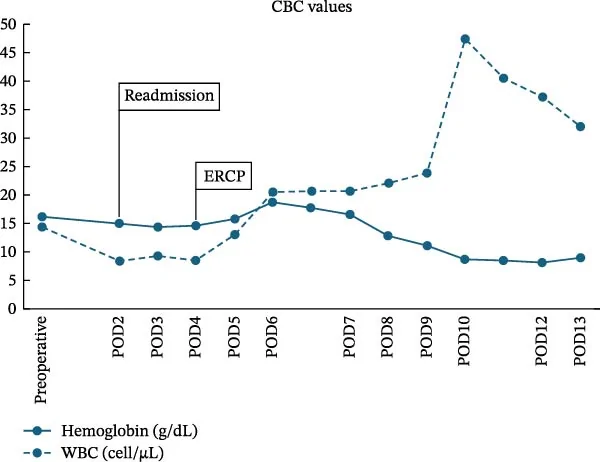
Blood counts throughout the initial hospital course.

The patient was readmitted and gastroenterology was consulted. An MRCP was ordered upon admission. However, in discussion with gastroenterology, due to the degree of hyperbilirubinemia (total bilirubin: 7.2 and direct bilirubin: 5.4) and continued increase in direct bilirubin after readmission, it was recommended to proceed with endoscopic evaluation without further imaging. He underwent an endoscopic ultrasound and endoscopic retrograde cholangiopancreatography (ERCP) on postoperative day 4, which was notable for an intact biliary tree without filling defects or biliary leak. A biliary sphincterotomy was performed for possible papillary stenosis; however, there was low suspicion for an obstructive etiology. A representative image is shown in Figure 2. Post‐procedure, the patient’s bilirubin continued to increase until it peaked on postoperative day 7 at a total bilirubin level of 14.1 with a direct component of 10.4. Due to the prolonged and persistent hyperbilirubinemia after ERCP, repeat imaging with an IV contrast CT abdomen/pelvis and MRCP were obtained, which demonstrated new evidence of acute pancreatitis without biliary abnormality or obstruction on MRI. Figures 3 and 4 demonstrate laboratory values including blood count and hepatic function panel throughout the patient’s initial hospital course. Over the following days, the patient’s bilirubins gradually declined without further intervention.

However, the patient’s course was further complicated by the development of severe ERCP‐induced necrotizing pancreatitis and delayed post ERCP bleeding requiring ICU admission, three image‐guided drain placements, bilateral video‐assisted retroperitoneal debridement, percutaneous endoscopic gastrojejunostomy tube placement, endoscopic cystogastrostomy, and multiple endoscopic necrosectomies. The patient was eventually discharged after a 4‐month hospital course, tolerating a limited oral diet, in stable condition.

## 3. Discussion

Abnormalities in postoperative liver function tests are a common occurrence after abdominal surgery, though the majority of liver enzyme elevations are mild and transient. Postoperative jaundice occurs in less than 1% of these cases [4]. Most patients who develop jaundice have underlying liver disease or cardiovascular disease, with the additional stress of surgery precipitating worsened hepatic function. Postoperative jaundice is believed to be multifactorial, related to bilirubin overproduction, blood reabsorption and decreased hepatic perfusion. Critically ill status, anesthetic drug choice, blood transfusion, and prolonged intraabdominal surgery times can also contribute.

Benign postoperative intrahepatic cholestasis has been described as a progressive elevation of bilirubin levels and resolution over the course of the first few postoperative weeks [1]. Elevations typically occur within the first 2–10 days after surgery as progressive conjugated hyperbilirubinemia. At their peak, these levels could be markedly elevated, with total bilirubin upward of 40 mg/dL. Of note, cases described commonly have involved patients who had complicated postoperative courses, concomitant sepsis, or required significant blood transfusion [4, 5]. In comparison, our patient was a healthy, young male with no comorbidities, who underwent a routine, uncomplicated surgery without undue physiologic stress.

Following laparoscopic cholecystectomy, studies have shown variant levels of transient mild liver enzyme elevations after uncomplicated elective surgery [6–9]. However, a majority of these studies did not note hyperbilirubinemia, and those patients that did demonstrate elevated bilirubin levels had mild elevations of primarily unconjugated bilirubin. It is posited that this transient elevation in unconjugated bilirubin is due to the negative impact of pneumoperitoneum on hepatic perfusion. Studies have shown elevations in markers of oxidative stress, validating an ischemia–reperfusion model [2, 3]. In our patient, however, we saw primarily conjugated hyperbilirubinemia, more typical of an obstructive or truly cholestatic pattern and not well explained by this model.

After cholecystectomy, in patients who present with hyperbilirubinemia, the differential diagnosis most commonly includes retained stones, bile leak, or bile duct injury and stricture. In a prospective study conducting routine cholangiography in patients undergoing laparoscopic cholecystectomy, common bile duct stones were found in approximately 3% of patients. However, not all patients are symptomatic or require retrieval of the stones with endoscopy, with around 2% of patients undergoing ERCP [10, 11]. The time frame for symptomatic retained common bile duct stones ranged from multiple weeks to years before presentation. Of the extrahepatic causes of hyperbilirubinemia after cholecystectomy, the most feared complication is bile duct injury. This can present with jaundice in the short term due to bile leakage or in the long‐term due to the development of bile duct strictures. Studies have shown the incidence of bile duct injuries to be less than 1% in laparoscopic cholecystectomy, with lower incidences in hospitals with greater experience [12]. Due to the increased ability to recognize aberrant anatomy, duct injuries, and common bile duct stones, recently, SAGES guidelines have shifted to recommend routine use rather than selective use of an IOC [13]. Because our patient underwent routine IOC, there was lower suspicion for retained common bile duct stone or missed biliary injury.

For these complications, management is most commonly with ERCP. However, ERCP is associated with its own morbidity. Complications can be common and severe, such as in this case. Severe complications occur in approximately 2% of patients. Common complications include both post‐sphincterotomy bleeding and ERCP‐induced pancreatitis, which occurs in approximately 3% of patients. Approximately 10% of pancreatitis cases are severe [14]. In the case of our patient, given that no direct complication was found to be due to the operation, the majority of their severe morbidity and prolonged course was secondary to the ERCP, which could have potentially been avoided with appropriate recognition of postoperative cholestasis as a driver of his lab abnormalities. While a passed retained stone remains a possibility and difficult to disprove retrospectively, the negative IOC and endoscopic findings in combination with the continued uptrend in bilirubins post‐ERCP suggest a lower likelihood.

Another consideration in this case is the possibility of ceftriaxone‐induced cholestasis or pseudolithiasis contributing to the patient’s hyperbilirubinemia. While drug‐induced liver injury is a common differential in patients with abnormal hepatic function tests, it can be a difficult diagnosis to establish. While the liver injury can manifest as direct hepatocellular damage and hepatitis, cholestasis as the result of abnormal biliary secretion is another common manifestation [15]. Of medications that can induce liver injury, one of the most common categories and widely used are antibiotics. These drug reactions can be idiosyncratic and can occur with as little as a single dose. Additionally, these reactions can be delayed after drug exposure [16]. Cases have described both the formation of biliary sludge and pseudolithiasis in response to ceftriaxone, which can cause hyperbilirubinemia or imaging findings mimicking cholelithiasis, which resolve with the discontinuation of the medication [17, 18]. In our patient, given the short timing between acute cholecystitis diagnosis and surgery, their antibiotic exposure was limited to a single dose of intravenous ceftriaxone and metronidazole. On readmission, there was no reexposure to these agents given the benign exam and lack of leukocytosis. However, given the idiosyncratic nature of these reactions, it is difficult to disprove retrospectively that hyperbilirubinemia is not attributable to a drug reaction.

Overall, this case differs from prior reports due to the suspected occurrence of postoperative intrahepatic cholestasis in an otherwise healthy, young patient after a routine, uncomplicated surgery. In this patient, due to the degree of direct hyperbilirubinemia, ERCP was pursued as the initial intervention, without further biliary imaging, due to concern for potential surgical biliary complications. One consideration is whether a pre‐procedure MRCP would have impacted this patient’s clinical course, given his complication burden post‐ERCP. In the case of this patient, this decision was made after discussion with gastroenterology. However, he had remained clinically stable and asymptomatic up until the ERCP and would not have been negatively impacted by the delay in the procedure to obtain further imaging. However, even with an unremarkable MRCP, given the continued uptrend in his bilirubin levels over the coming days after ERCP, it is likely that he still would have undergone further endoscopic intervention. While surgical complications should remain the highest on the differential in a postoperative patient after laparoscopic cholecystectomy, it is important to be aware of alternative more benign causes of hyperbilirubinemia in postoperative patients, which can cause severe bilirubin elevations. This case may prove a cautionary tale for those managing patients presenting with hyperbilirubinemia following routine minimally invasive cholecystectomy.

## Funding

No funding was received for this study.

## Conflicts of Interest

The authors declare no conflicts of interest.

## Data Availability

Data sharing is not applicable to this article, as no datasets were generated or analyzed during the current study.
